# Oxidative stress‐induced FAK activation contributes to uterine serous carcinoma aggressiveness

**DOI:** 10.1002/1878-0261.13346

**Published:** 2022-12-07

**Authors:** Isabel C. Lopez‐Mejia, Jordi Pijuan, Raúl Navaridas, Maria Santacana, Sònia Gatius, Ana Velasco, Gerard Castellà, Anaïs Panosa, Elisa Cabiscol, Miquel Pinyol, Laura Coll, Núria Bonifaci, Laura Plata Peña, August Vidal, Alberto Villanueva, Eloi Gari, David Llobet‐Navàs, Lluis Fajas, Xavier Matias‐Guiu, Andrée Yeramian

**Affiliations:** ^1^ Center for Integrative Genomics University of Lausanne Switzerland; ^2^ Laboratory of Neurogenetics and Molecular Medicine – Pediatric Institute of Rare Diseases Institut de Recerca Sant Joan de Déu Barcelona Spain; ^3^ Departament de Ciències Mèdiques Bàsiques, IRBLleida University of Lleida Spain; ^4^ Pathology Group, Department of Pathology and Molecular Genetics, Hospital Universitari Arnau de Vilanova (HUAV), IRBLleida University of Lleida Spain; ^5^ Biostatistics Unit, Hospital Universitari Arnau de Vilanova, IRB‐Lleida University of Lleida Spain; ^6^ Flow Cytometry and Confocal Microscopy Unit, IRBLleida University of Lleida Spain; ^7^ Molecular Mechanisms and Experimental Therapy in Oncology‐Oncobell Program, Bellvitge Biomedical Research Institute (IDIBELL) Gran via de l'Hospitalet Barcelona Spain; ^8^ Centro de Investigación Biomédica en Red de Cáncer (CIBERONC) Instituto de Salud Carlos III (ISCIII) Madrid Spain; ^9^ Department of Pathology‐Hospital Universitari de Bellvitge Barcelona Spain; ^10^ Xenopat S.L., Parc Cientific de Barcelona (PCB) Spain; ^11^ Program Against Cancer Therapeutic Resistance (ProCURE), ICO, IDIBELL Barcelona Spain

**Keywords:** focal adhesion kinase, migration, reactive oxygen species, uterine serous carcinoma

## Abstract

Uterine serous carcinoma (USC) is an aggressive form of endometrial cancer (EC), characterized by its high propensity for metastases. In fact, while endometrioid endometrial carcinoma (EEC), which accounts for 85% of EC, presents a good prognosis, USC is the most frequently fatal. Herein, we used for the first time a peptide‐based tyrosine‐kinase‐activity profiling approach to quantify the changes in tyrosine kinase activation between USC and EEC. Among the tyrosine kinases highly activated in USC, we identified focal adhesion kinase (FAK). We conducted mechanistic studies using cellular models. In a USC cell line, targeting FAK either by inhibitors PF‐573228 and defactinib (VS‐6063) or by gene silencing limits 3D cell growth and reduces cell migration. Moreover, results from our studies suggest that oxidative stress is increased in USC tumors compared to EEC ones. Reactive oxygen species (ROS) induce tyrosine phosphorylation of FAK and a concomitant tyrosine phosphorylation of paxillin, a mediator of FAK signal transduction. Mechanistically, by tracking hundreds of individual cells per condition, we show that ROS increased cell distance and migration velocity, highlighting the role of ROS‐FAK‐PAX signaling in cell migration. Both defactinib and ROS scavenger N‐acetylcysteine (NAC) revert this effect, pointing toward ROS as potential culprits for the increase in USC cell motility. A proof of concept of the role of FAK in controlling cell growth was obtained in *in vivo* experiments using cancer‐tissue‐originated spheroids (CTOS) and a patient‐derived orthotopic xenograft model (orthoxenograft/PDOX). Defactinib reduces cell proliferation and protein oxidation, supporting a pro‐tumoral antioxidant role of FAK, whereas antioxidant NAC reverts FAK inhibitor effects. Overall, our data points to ROS‐mediated FAK activation in USC as being responsible for the poor prognosis of this tumor type and emphasize the potential of FAK inhibition for USC treatment.

## Graphical abstract

Patients with uterine serous carcinoma (USC) have reduced overall survival. Here, using the Pamgene multiplex kinase activity assay, we demonstrate that focal adhesion kinase (FAK) is activated in USC, and that oxidative stress is a main activator of this kinase. FAK activation cascade promotes tumor growth and migration, while its inhibition reduces tumor growth *in vitro* and *in vivo*, in part, by increasing oxidative stress.

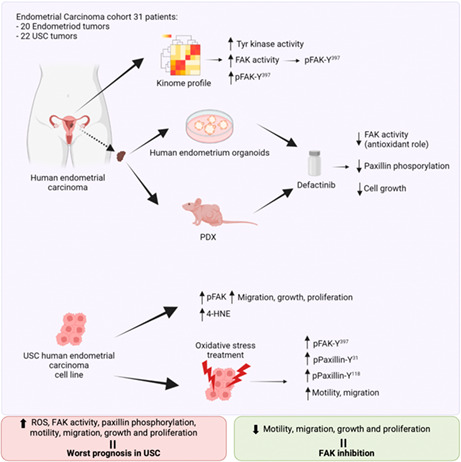

**Abbreviations**
BrdU: bromodeoxyuridine
CTOS: cancer‐tissue‐originated spheroids
DMEM: Dulbecco's modified Eagle's Medium
DNP: anti‐dinitrophenyl
DNPH: 2,4‐dinitrophenylhydrazine
EC: endometrial carcinoma
EEC: endometrioid endometrial carcinoma
FAK: focal adhesion kinase
MSI: microsatellite instability
NAC: N‐acetylcysteine
PDOX: patient‐derived orthotopic xenograft
ROS: reactive oxygen species
scr: scramble
TCGA: the cancer genome atlas
TMA: tissue micro array
USC: uterine serous carcinoma

## 1 Introduction

Endometrial carcinoma is the fifth most diagnosed gynecological malignancy worldwide [1] and ranks as the 14^th^ cause of cancer‐related deaths in women. In the group of endometrial cancers, uterine serous carcinomas (USC) account for approximately 10% of corpus cancer cases [2], and present the poorest prognosis, with 5‐year survival rates as low as 55% [2], in contrast to the hormone‐responsive endometrioid endometrial carcinomas (EEC) which represent almost 85% of endometrial carcinomas and present a better prognostic. The Cancer Genome Atlas (TCGA) studies based on exome sequence, epigenomic and transcriptomic data have divided endometrial cancers in four molecular different subgroups: polymerase epsilon (*POLε*) ultra‐mutated, microsatellite instability (MSI) hyper‐mutated, EC with low copy number alterations and high copy‐number tumors [3]. This latest group is mostly composed of USC, but also includes some EEC of mainly grade 3. As USC exhibit a worse therapeutic outcome than EEC, we believe that a better understanding of the biology of this cancer is urgently needed to identify new therapeutic options.

Protein tyrosine kinases control a wide range of biological processes and their uncontrolled activation is a common feature of many cancers [4], rendering them potential biomarkers for targeted therapy. In this work, we have compared global tyrosine kinase activity profiles of patients' tumors of both endometrioid (EEC) and serous (USC) histology using the peptide array system [5, 6] developed by Pamgene, with the aim of identifying potential biomarkers for targeted therapy. We identified focal adhesion kinase (FAK), as a putative upstream kinase showing higher activation levels in serous *vs*. endometrioid cancer samples. FAK is a cytosolic tyrosine kinase that is canonically activated in the integrin‐rich adhesion sites known as focal contacts upon engagement of the extracellular matrix with integrin receptors [7]. FAK is encoded by the *PTK2* gene that can be amplified in cancers of the female reproductive system including endometrial cancer [8]. Its activation is characterized by auto‐phosphorylation at Tyrosine 397 (Y^397^), a hallmark of FAK activity [9] which serves as a binding site for various SH2‐domain‐containing proteins, including Src kinases, which then phosphorylate FAK at Y^576^ and Y^577^ leading to its full activation [10], and also various downstream targets such as paxillin and p130Casn which mediate cell–cell adhesion contacts and cell motility [11]. Paxillin is a cytoskeletal component of the focal contacts and is phosphorylated by FAK‐Src on Y^31^ and Y^118^, enabling focal contacts turnover [12], cell motility [13], and FA (focal adhesions) disassembly [14]. Moreover, in the context of tumor biology, FAK has been shown to regulate cell migration and adhesion, promote tumor growth and metastasis, as well as facilitate cell survival in stress conditions [9, 15, 16, 17]. Herein, following the peptide‐based approach, we have assessed FAK activation levels by western blot in 28 biopsies from USC (13 biopsies) and EEC (15 biopsies), confirming the results obtained by the peptide array. We next evaluated the therapeutic effects of FAK targeting either by using pharmacological inhibitors [PF‐573228 and defactinib (VS‐6063)] or by silencing FAK through shRNA, in USC cells *in vitro*. Moreover, in the last years, reactive oxygen species (ROS) have also emerged as important short‐lived messengers that act on a wide range of physiological processes, including different transduction pathways which influence actin cytoskeleton dynamics promoting changes in cell motility and cell adhesion [18, 19, 20] and modifying the distribution of FAK in focal contact sites [21]. Interestingly, integrin engagement has been shown to generate intracellular reactive oxygen species (ROS) that lead to FAK activation and cell adhesion [18]. We thus analyzed the interplay between ROS and FAK activation in USC. We show that oxidative stress markers are enhanced in USC and that ROS activate FAK pathway promoting migration and growth of this tumor type. Mechanistically, we demonstrate that targeting FAK significantly decreases USC cell growth and migration and that this effect is completely reversed by ROS scavenger, pointing to an antioxidant protective role of FAK. Altogether our findings establish FAK and ROS as previously unsuspected key players in USC tumorigenesis and reveal a novel mechanism used by ROS to induce USC cell growth and migration.

## 2 Materials and methods

To gain a deeper knowledge of global tyrosine kinase activity profiles of tumors of both endometrioid (EEC) and serous (USC) histology, we have processed patients' samples after obtaining their consent as explained below. We conducted a peptide‐based tyrosine kinase array screen, western blot and immunohistochemical studies on these samples and FAK kinase emerged as a top hit kinase that showed higher activation levels in USC samples. Next, FAK inhibition (pharmacologically and shRNA‐lentiviral‐mediated silencing) was tested in 3D cell growth and cell migration models. *In vivo* experiments using cancer tissue‐originated spheroids and a patient‐derived orthotopic xenograft model were conducted to support the results obtained *in vitro*.

### 2.1 Ethics statement and patient samples

All the procedures were carried out in accordance with the Helsinki declaration, after obtaining the approval of the IRBLleida Biobank Ethic Committee (Approval number 1892) and the Bellvitge university hospital (approval number PR047/18). Informed written consent was obtained from patients. Endometrial cancer samples were obtained by surgical resection and, were snap‐frozen in liquid N_2_ and stored at −80 °C.

### 2.2 Patient samples processing, classification, and TMA construction

Endometrial cancer samples were obtained from the Pathology department of the Hospital Universitari Arnau de Vilanova (HUAV), Lleida, Spain and divided into two sections by the pathologist, corresponding to the superficial and deep parts (the deep part corresponds to the invasive front) of the tumor sample. Samples were grouped in EEC group and USC based on histological and morphological features [22], and also according to the new classification system [3] based on the TCGA surrogate which applies a diagnostic algorithm using three immunohistochemical markers (MSH6, PMS2, and p53) [23] and one molecular test (mutation analysis of the exonuclease domain of POLε) [24]. Expression analysis of the marker genes p53, MSH6 and PMS2 was assessed by immunohistochemistry in FFPE (Formalin‐fixed paraffin‐embedded) tissue material, using the following antibodies: p53 (clone DO‐7), MSH6 (clone EP49), PMS2 (clone EP51) from Dako (Agilent Technologies‐DAKO, Santa Clara, CA, USA). Mutations analysis of POLε exonuclease domain coded by exons 9, 11, 13, and 14 was assessed by primer‐specific PCR amplification and Sanger sequencing [23, 24]. Four tissue microarrays (TMAs) from EEC and USC tumor biopsies/curettages were constructed using an automated Tissue Micro Arrayer (TMA Grand Master, 3D HISTEC, Budapest, Hungary).

### 2.3 Antibodies and reagents

The antibodies used were as follows: pFAK‐Y^397^ (Thermo Fisher Scientific, Waltham, MA, USA; rabbit monoclonal, clone 31H5L17). Of note p‐FAK‐Y^397^ antibody specificity was assessed in Supplementary Information. Total FAK (Santa Cruz Biotechnology, Dallas, TX, USA; mouse monoclonal, clone D1, sc‐271 126), pPaxillin‐Y^31^ (Thermo Fisher Scientific; rabbit polyclonal, 44‐720G), pPaxillin‐Y^118^ (Cell Signaling, Danvers, MA, USA; rabbit polyclonal, 2541), β‐actin (Sigma‐Aldrich, St Louis, MO, USA; mouse monoclonal, clone AC‐15, A5441), 4‐HNE (Abcam, Cambridge, rabbit polyclonal, UK; ab46545), and HIF‐1α (BD Biosciences, San Diego, CA, USA; mouse monoclonal, clone 54/HIF‐1α, 610 958). Anti‐dinitrophenyl (DNP) (goat polyclonal, A150‐117A) was from Bethyl, while rhodamine‐conjugated phalloidin (P1951) and α‐tubulin (mouse monoclonal, clone B‐5‐1‐2) were from Sigma‐Aldrich. Cleaved caspase 3 antibody was purchased from Cell Signaling (rabbit polyclonal, 9661) and BrdU antibody was from Invitrogen (Carlsbad, CA, USA; mouse monoclonal, clone BU20A, 14‐5071‐82). Peroxidase conjugated goat anti‐mouse (115‐035‐003) and goat anti‐rabbit (111‐035‐003) antibodies (Jackson Immunoresearch, West Grove, PA, USA), and secondary anti‐rabbit Alexa Fluor 488 (Thermo Fisher Scientific; A11008). PF‐573228 (PZ0117) and H_2_O_2_ (95294‐1L) were purchased from Sigma‐Aldrich. Matrigel was from BD Biosciences (354234) and defactinib (VS‐6063, PF‐04554878) was purchased from Selleckchem (Houston, TX, USA). DMEM‐F12 medium was from Sigma‐Aldrich; 11 580 376 and dextran‐coated charcoal‐stripped serum from HyClone Laboratories (11571821) Inc., Logan, UT, USA.

### 2.4 Cell lines and cell culture

ARK‐1 (RRID: CVCL_IV72) primary human uterine serous carcinoma cell line was provided by Dr. Alessandro D. Santin (Yale University, New Haven, CT, USA) [25]. Ishikawa 3‐H‐12 cell line (IK, RRID: CVCL_2529), was obtained from the American Type Culture Collection (ATCC, Manassas, VA, USA). Ishikawa cell line was authenticated by short tandem repeat (STR) analysis, yielding more than 80% match in profiled loci. The STR analysis was also done for ARK‐1 cells, but no appropriate STR reference was found in the public database. Cells lines were grown in Dulbecco's modified Eagle's Medium (Sigma‐Aldrich) supplemented with 10% Fetal Bovine Serum (FBS) (Invitrogen), 1 mm HEPES (Sigma‐Aldrich), 2 mm L‐Glutamine (Sigma‐Aldrich) 1 mm sodium pyruvate (Sigma‐Aldrich), and 1% of penicillin/streptomycin (Sigma‐Aldrich) at 37 °C with saturating humidity and 5% CO_2_. All experiments with cell lines were performed in mycoplasma‐free cells.

### 2.5 3D spheroid culture, bromodeoxyuridine (BrdU) incorporation

3D spheroid cultures were performed either from USC‐derived tumor tissue or EC cell lines. For 3D cultures from patient‐derived tumor tissue (USC‐END266), the culture was performed as described previously [26], with minor modifications. Briefly, cells were diluted in DMEM‐F12 medium containing 2% dextran‐coated charcoal‐stripped serum (DCC). Cells were plated on Matrigel‐coated wells and were cultured for 2–7 days in an incubator at 37 °C with saturating humidity and 5% CO_2_. At the indicated times, spheroid phase‐contrast was imaged, and glandular perimeter or diameter was determined using image j analysis software (imagej v.1.46r; NIH, Bethesda, MD, USA). For each experiment, at least 100 spheroids were quantified. For BrdU experiments, 48 h post‐seeding, cells were pulsed with 83 μg·mL^−1^ BrdU (Sigma‐Aldrich; B5002) for 15 h and then fixed with 4% paraformaldehyde (PFA). DNA was then denatured with 2 mol·L^−1^ HCl for 30 min and neutralization was achieved with 0.1 mol·L^−1^ Na_2_B_4_O_7_ (pH 8.5) for 2 min. Cells were then blocked with a solution containing 5% horse serum, 5% FBS, 0.2% glycine, and 0.1% Triton X‐100 for 1 h. Next, cells were incubated with a mouse anti‐BrdU monoclonal antibody (1 : 100; Invitrogen, BU20A) and Alexa Fluor‐conjugated anti‐mouse secondary antibody. Nuclei were counterstained with 5 μg·mL^−1^ Hoechst 33258, and cells were visualized under a confocal microscope. BrdU‐positive nuclei were counted and divided by the total number of cells (visualized by Hoechst staining). The results are expressed as a percentage of BrdU‐positive cells.

### 2.6 Lentivirus packaging and infection and generation of stably transduced ARK‐1 cells

Lentiviral‐based vectors for RNA interference gene silencing were pLKO.1‐puro (Sigma‐Aldrich), containing either shRNAs or scrambled sequences. FAK shRNA lentiviral vectors were purchased from Sigma (Mission shRNA, TRCN0000196310). Lentiviruses were produced by co‐transfecting HEK 293T packaging cells as described previously [27]. Stable knockdown‐resistant cells were selected with Puromycin (2.5 μg·mL^−1^).

### 2.7 Western blot analysis

Endometrial adenocarcinoma cell lines and tumor samples were lysed with lysis buffer [2% SDS, 125 mm Tris–HCl (pH 6.8)] under native conditions with M‐PER buffer (M‐PER Mammalian Extraction Buffer, Pierce, Rockford, IL, USA), respectively, supplemented with proteases and phosphatases inhibitors, and subjected to western blotting as described in previous work [28]. Protein concentrations were determined with the Protein Assay kit (Bio‐Rad, Hercules, CA, USA). Equal amounts of proteins were subjected to SDS/PAGE and transferred to PVDF membranes (Millipore, Bedford, MA, USA). Membranes were blocked in T‐BST (20 mm Tris–HCl pH 7.4, 150 mm NaCl, 0.1% Tween‐20) plus 3% of bovine serum albumin (BSA), and then incubated with the specific primary antibodies overnight at 4 °C and with peroxidase‐coupled anti‐mouse or anti‐rabbit secondary antibodies for 1 h followed by chemiluminescent detection with ECL Advance (Amersham‐Pharmacia, Buckinghamshire, UK). Chemiluminescence signal was visualized using Chemi Doc MP imaging system, and images were analyzed by the Image Lab software (Life Science Research, Bio‐Rad). Analysis of protein oxidation pattern was performed by derivatization of carbonyl groups with 2,4‐dinitrophenylhydrazine (DNPH), as described previously [29] with minor modifications. Cells were lysed with NP‐40 buffer with protease and phosphatase inhibitors, and protein concentration was determined with the Protein Assay kit (Bio‐Rad) as stated earlier. Twenty micrograms of total protein was collected for each condition, and SDS was added to the samples to a final concentration of 6%. Samples were then heated at 95 °C for 2 min and centrifuged at 2380 ***g*** for 5 min. Twenty microliters of the recovered supernatant was then mixed with 20 μL of 10 mm DNPH in 10% trifluoroacetic acid. The reaction was left for 10 min at 25 °C and was stopped by the addition of 20 μL of 2 m Tris, 30% glycerol, and 15% β‐mercaptoethanol. Samples were then loaded on SDS‐polyacrylamide gel electrophoresis (SDS/PAGE) and screened with anti‐dinitrophenyl (DNP) antibody.

### 2.8 Generation of orthoxenograft of USC in mice

The USC orthoxenograft was developed in nude mice by orthotopic implantation of a small fresh fragment of a primary USC surgical specimen (USC‐END266) obtained at Hospital Universitari de Bellvitge (Hospitalet de Llobregat, Barcelona, Spain) with the informed consent of the patient, as we described previously [30]. Briefly, a tumor sample taken by pathologist was placed in DMEM supplemented with 10% FCS and penicillin/streptomycin and implanted in two 5‐week‐old female NOD‐*scid* IL2Rγ^null^ (NSG) mice weighing 18–22 g. Mice were anesthetized by isoflurane inhalation, subjected to a lateral laparotomy, and a small fragment of 4 × 4 mm^3^ maintaining tridimensional structure was anchored in the uterus with a Prolene 7.0 suture. Once the tumor was grown, (USC‐END266) the mouse was sacrificed, and tumor was re‐implanted in another three mice to expand and generate enough high‐quality tissue for seeding drug assay experiment. A good histology correlation was evidenced between primary and orthotopic xenografted tumors.

Then, 15 mice were orthotophically implanted with USC‐END266 at passage #2 and 60 days later when homogeneous small masses were detected at palpation, 14 mice were randomized and allocated into two treatment groups: (a) Vehicle solution (*n* = 5 mice) and (b) VS‐6063 (100 mg·kg^−1^) (*n* = 9 mice). VS‐6063 was resuspended in a solution containing 5% DMSO, 50% PEG300, and 5% Tween‐80 and animals were treated daily by oral gave for 4 weeks. At sacrifice, tumors were collected, photographed, and measured. Diaphragm, liver, lung, and peritoneal cavity were macroscopically inspected for the presence of metastases, and the different organs were collected to detect the presence of micrometastases by standard hematoxylin–eosin analysis.

Animals were housed in a sterile environment, in cages with autoclaved bedding, food, and water and maintained on a daily 12 h' light, 12 h' dark cycle. The Institutional Ethics Committees approved the study protocol, and the animal experimental design was approved by the IDIBELL animal facility committee (AAALAC Unit1155) under approved procedure 9111. All experiments were performed in accordance with the guideline for Ethical Conduct in the Care and Use of Animals as stated in The International Guiding Principles for Biomedical Research Involving Animals, developed by the Council for International Organizations of Medical Sciences.

### 2.9 Tyrosine kinase activity profiling using PamChip ^®^ peptide microarrays, and upstream kinase analysis

**Fig. 1:**
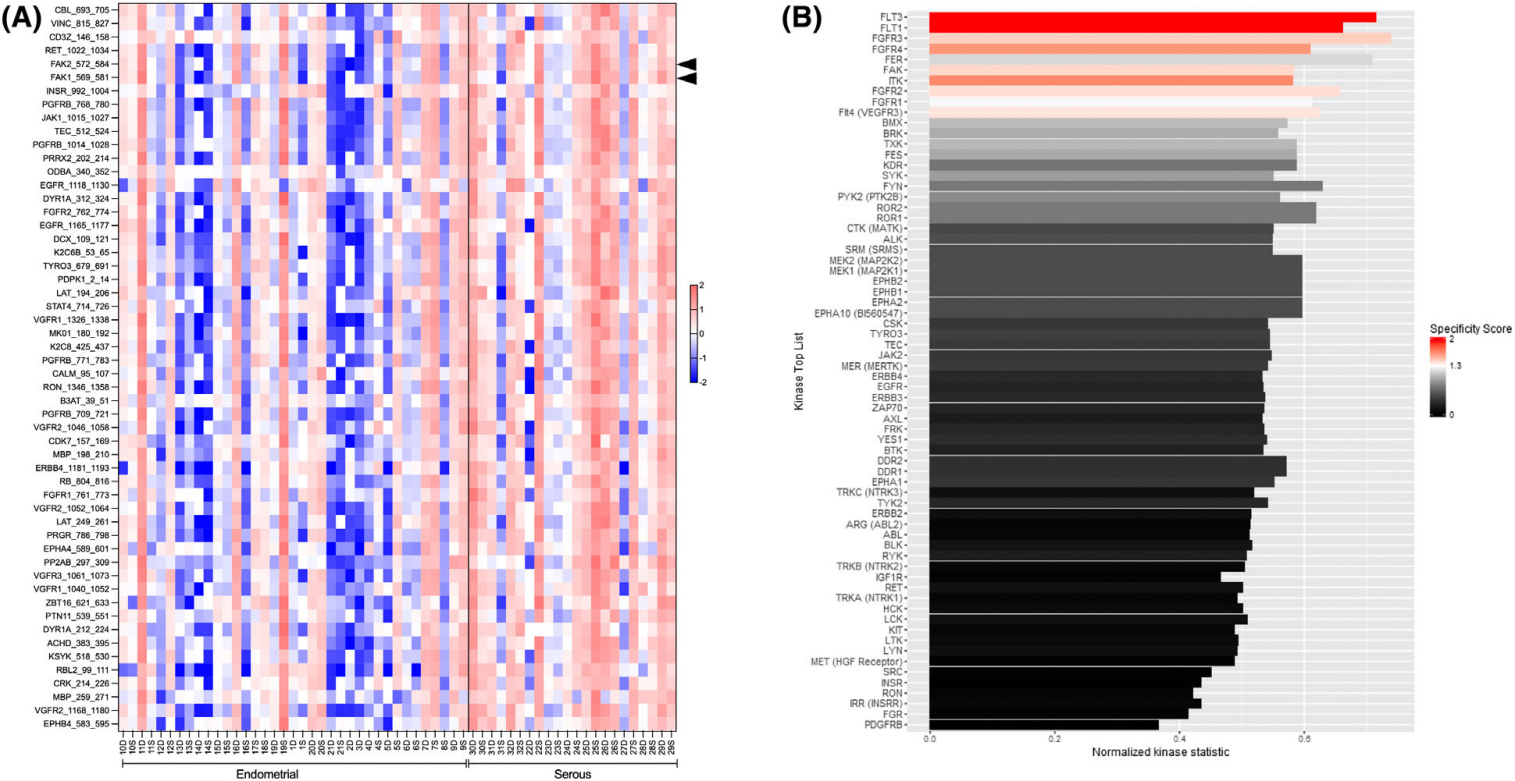
Identification of putative tyrosine kinases with differential activities in EEC *vs*. USC tumor samples. (A) Forty EEC and 22 USC tumor samples were analyzed for tyrosine kinase activity. Equal amounts of tumor protein lysates were analyzed on PTK peptide microarrays. The PTK experiments were performed using a Pamstation PS12 in more than five independent runs with an equal number of samples from each experimental group. Peptide phosphorylation was analyzed using the Bionavigator software. Signal intensities of the phosphorylated peptides were monitored and represented as a z‐scored data. The heat‐map shows the 53 peptides with significant differences in phosphorylation between both groups when compared by *t*‐test (*P < 0.05). The increase in red color highlights an overall increase in kinase activity in USC samples compared to EEC ones. Of note, FAK1 and FAK2 peptides are highlighted by arrowheads in the figure. (B) List of putative upstream kinases with differential activity between USC and EEC samples. Putative upstream analysis was performed using the bionavigator software ‘Upstream PTK’ module. The length of the bars corresponds to the normalized kinase statistic, a positive value of the kinase statistic indicates higher activity in USC samples. The color of the bars denotes specificity score. Of note, positive kinase statistics means USC samples show higher tyrosine kinase activity compared to EEC ones.

Tyrosine kinase activity profiles were determined using the PamChip^®^ peptide tyrosine kinase microarray system on PamStation^®^12 (Pamgene, BJ's‐Hertogenbosch, The Netherlands), according to the manufacturer's protocol. For protein sample preparation, tumor samples were grinded into powder in liquid N_2_ with precooled ceramic mortar and pestles. Tumor lysates were obtained by lysing tumors in M‐PER lysis buffer (Mammalian Protein Extraction Buffer, Pierce), supplemented with 1 : 50 Halt Phosphatase Inhibitor cocktail (Thermo Fisher Scientific) and 1 : 50 Halt Protease inhibitor cocktail EDTA‐free (Thermo Fisher Scientific), for 20 min on a wheel at 4 °C. Lysates were then centrifuged for 10 min at 4 °C at 16089 ***g*** to remove all debris. The supernatant was transferred aliquoted into new precooled tubes and snap‐frozen in liquid N_2_, one aliquot was used to measure protein concentration using the Pierce BCA protein assay. The remaining aliquots were stored at −80 °C until further processing. Equal amounts of EEC and USC protein samples were loaded on peptide PTK microarrays. Each PamChip array harbors 144 peptides or kinase substrates spotted on its matrix. Phosphorylation of the peptides or substrates was monitored by the signal emitted by the FITC labeled anti‐phosphotyrosine PY‐20 antibody, and the intensity signal was recorded by a CCD (charged coupled device)‐camera in the PamStation. The images obtained by the camera were then quantified using the supplied Package BioNavigator software (Pamgene International BV). A signal‐to‐background threshold was used to select 106 peptides for downstream analysis. Peptides that showed significant phosphorylation change between EEC and USC samples and the corresponding putative Upstream kinases were determined using the ‘two group comparison’ and ‘Upstream PTK’ pipeline (based on *in silico* motif predictions from the phosphonet kinexus database (PhosphoNet, http://www.phosphonet.ca/)) from the Bionavigator software, respectively. Upstream kinase prediction analysis was done considering the top‐five ranked kinases for each substrate The list of peptides showing significant differences in phosphorylation between the two groups is shown in Fig. 1A. The list of the upstream kinases differently activated between the two types of tumor samples is shown in Fig. 1B.

### 2.10 Single‐cell live‐cell imaging and kymograph analysis

For cell tracking experiments (single cell), 3000 cells were seeded in a 96 multi‐well plate and left to attach. Twenty‐four hours later, cells were either stimulated with H_2_O_2_ and/or defactinib or left untreated. Experiments were performed in a Zeiss Axio Observer Z1 microscope using a 20× objective and imaging every 20 min for 20 h as previously described [31]. Single cells at the start of the time series, remaining within the ROI and not dividing during the time series were selected and tracked. Cell tracking was performed using the Plugin ‘Manual Tracking’ in the ImageJ software, selecting the center of the cell nucleus at each time point during the time series. Tracking analysis was performed by exporting the tracks into Ibidi's ‘Chemotaxis and Migration’ tool. Migration parameters [velocity (μm·min^−1^), accumulated distance (μm), and trajectory (μm)] were plotted.

### 2.11 Immunohistochemistry and immunofluorescent staining

Immunohistochemical staining was performed as described previously [28]. For immunofluorescence staining cells were visualized and analyzed using confocal microscopy (FV1000; Olympus, Tokyo, Japan) with the ×10 and the oil‐immersion ×60 magnification objectives. Cells were seeded in 96‐well black plates with a microclear bottom (Greiner Bio‐One, Stonehouse, UK; 655 090) or seeded for 3D spheroid culture. Cells were fixed with 4% PFA, permeabilized with 0.1% Triton X‐100, blocked [2% horse serum, and 0.1% Triton X‐100, 2% BSA in phosphate‐buffered saline (PBS)] and were incubated with the specific primary and secondary antibodies. The images were acquired with the same settings for all conditions. Analysis of images was obtained with fluoview fv1000 software (Olympus).

### 2.12 Statistical analysis

All data are expressed as mean ± standard deviation (SD) or box plots showing the median, box edges represent the [25^th^ and 75^th^ percentiles], and the whiskers extend to the minimum and maximum values. The specific statistic test applied in each case is indicated in the figure legend. *P*‐values are indicated by asterisks *P < 0.05, **P < 0.01, ***P < 0.001.

Statistical analysis computing and graphs were performed using the graphpad prism version 8.0.1 (GraphPad software Inc, La Jolla, CA, USA). The normality of data was assessed by the Kolmogorov–Smirnov test followed by *t*‐test or ANOVA and Tukey Kramer *post hoc* test (parametric test) or Mann–Whitney *U*‐test (nonparametric test). Mantel–Haenszel χ^2^ test was used to evaluate the potential differences in mean percentages of EEC *vs*. USC patients that died of metastasis. Correlation studies, with a logarithmic graphical scatter plot representation were performed to test the associations between p‐FAK‐Y^397^/β‐actin *vs*. DNP/β‐actin protein levels (Spearman rank). For survival analysis, the Gehan–Breslow–Wilcoxon test was used.

## 3 Results

### 3.1 Molecular classification of EEC and USC samples based on the TCGA database and their clinical outcome

The samples used in this study were classified into EEC and USC groups, based on histomorphological criteria and on the TCGA classification (Table S1). Survival analysis using Gehan–Breslow–Wilcoxon test revealed that USC patients had a statistically significant shorter overall survival after surgery compared to EEC patients (*P* = 0.002; Fig. S1). Herein, almost 5 years' post‐surgery (1824 days), 75% of EEC patients were still alive *vs*. 12.5% for USC patients. Of note, while 10% of EEC patients died of metastasis (2/20), this percentage reached 87.51% (6/7) in USC patients (*P* < 0.0001; Mantel–Haenszel χ^2^ test; Table S1).

### 3.2 Increased phosphorylation of PTK peptide substrates in USC biopsies compared to EEC

Tyrosine kinase activity profile was assessed using Pamgene technology in EEC and USC tumor samples obtained by surgical resection. During resection, and because of intra‐tumoral heterogeneity, each tumor was divided into two sections by the pathologist (Dr. Xavier Matias‐Guiu), corresponding to the superficial and deep parts (invasive front). Both samples were considered as independent samples in further analyses. Thus, from a total of 31 patients, we obtained 40 EEC samples (20 superficial and 20 deep samples), and 22 USC samples (11 superficial and 11 deep samples). Tumor lysates with sufficient protein concentration were used for the assay. After 1 h of kinase reaction, signal intensities of the phosphorylated peptides were monitored and represented as a *z*‐scored data. Increased phosphorylation for most tyrosine phosphorylated peptides was observed in USC samples compared to EEC ones. Peptides showing a significant difference between both groups, when compared by *t*‐test (*P* < 0.05) are shown in the heat map of Fig. 1A. Of note, significant higher phosphorylation intensity of a FAK1 (569‐RYMEDSTYYKASK‐581) peptide‐containing tyrosine 576 and 577 was registered in USC samples compared to EEC samples (USC: 6.79 *vs*. EEC: 6.19; *P* = 0.02 for FAK1). Similar results were obtained for a peptide from the FAK1 paralog FAK2 (572‐RYIEDEDYYKASV‐584) (USC: 8.40 *vs*. 7.82; *P* = 0.03 for FAK2).

### 3.3 Increased overall tyrosine kinase activity in USC samples compared to EEC samples

Tyrosine phosphorylated peptides were used to identify the putative tyrosine kinases responsible for the differences observed between USC and EEC samples (Fig. 1B, see Supplementary methods). The length of the bars corresponds to the kinase statistic for each of these kinases. A value > 0 indicates a higher activation level of a kinase in the USC group. There is a significant up‐regulation of the overall tyrosine kinase activity in USC samples compared to EEC (Fig. 1B). A significant activation of Flt (Flt‐3, Flt‐4, and Flt‐1), FAK and FGFR (FGFR‐3, and FGFR‐2) kinases is observed in USC samples compared to EEC, these kinases also ranked as the top kinases according to their final score (Fig. S2A) and their specificity score (Fig. S2B).

### 3.4 USC cell lines, tumors, and aspirates exhibit enhanced FAK activation

**Fig. 2:**
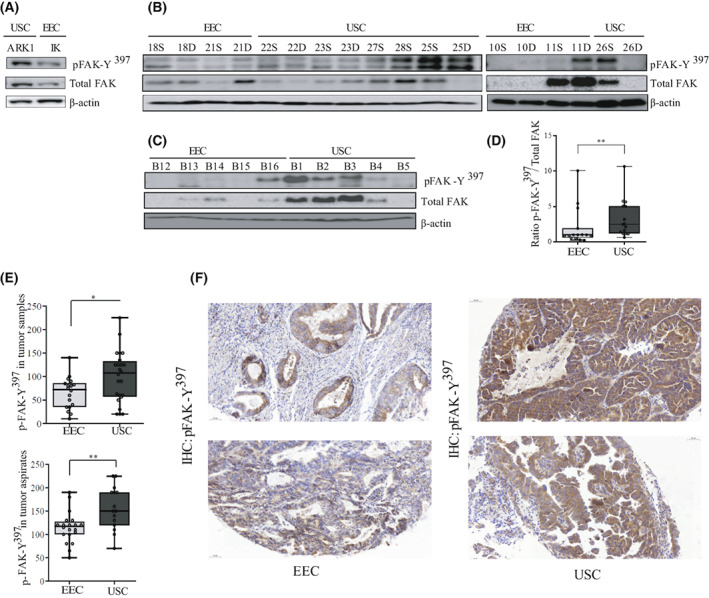
Enhanced FAK activation in USC cell lines, aspirates, and tumor samples compared to their counterparts. (A) Representative western blot analysis of p‐FAK‐Y^397^ and total FAK levels in ARK‐1 USC and Ishikawa (IK) EEC cell lines (*n* = 3 independent experiments). β‐actin was used as a loading control. (B) Representative western blot of tumor samples used for the Kinome analysis. A total of 18 EEC and USC samples corresponding to either the superficial part (S) or the deep (D) part of the tumor, were processed under native conditions and analyzed for p‐FAK‐Y^397^ and total FAK levels. *n* = 3 independent experiments. β‐actin was used as a loading control. (C) Ten independent samples corresponding to five EEC tumor samples and five USC samples were processed under native conditions to corroborate previous results. (D) Values of p‐FAK‐Y^397^/total FAK ratio levels between all EEC and USC samples used in B, presented as median ± CI (*n* = 3 independent experiments). Mann–Whitney *U*‐test (**P = 0.007). (E) p‐FAK‐Y^397^ staining in tumor samples and tumor aspirates was calculated by a histoscore method (being 250 points the maximum immunoreactivity), and values are median ± CI (*n* = 3 independent experiments). Statistical analysis was performed to assess differences in p‐FAK‐Y^397^ staining between EEC and USC tumors and aspirates using Mann–Whitney *U*‐test. Both USC tumors and aspirates show a significant higher expression of p‐FAK‐Y^397^ levels compared to their EEC counterparts (*P = 0.03 and **P = 0.006, respectively). (F) Representative images of p‐FAK‐Y^397^staining in two EEC and two USC samples. Scale bar: 50 μm, 20× magnification.

Based on the obtained results, and as aberrant FAK kinase activation controls multiple biological processes involved in cancer progression [32], we next aimed to check its role in USC. We first assessed total FAK levels and its activation (p‐FAK‐Y^397^) in two endometrial cancer cell lines. As shown in Fig. 2A, ARK‐1 USC cell line showed higher total FAK and p‐FAK‐Y^397^ levels compared to EEC Ishikawa (IK) cells, suggesting a possible amplification of FAK in this cell line. Next, we assessed total FAK and p‐FAK‐Y^397^ levels in a panel of 18 tumor samples that were previously used for the tyrosine peptide array (Fig. 2B). A validation experiment was also performed in an independent cohort of 10 tumor samples (5 EEC and 5 USC; Fig. 2C). Statistical analysis of p‐FAK‐Y^397^/total FAK relative levels in all analyzed samples confirmed significantly higher levels of FAK activation in USC tumors compared to EEC (Fig. 2D; p‐FAK‐Y^397^/total FAK ratio: 3.3 ± 2.6 in USC *vs*. 2.02 ± 2.7 in EEC samples; *P* = 0.007). Moreover, the intensity of p‐FAK‐Y^397^ staining was compared by immunohistochemistry in a set of EEC and USC tumor samples from the diagnosed endometrial aspirates and in another set of samples from hysterectomy specimens. Consistent with western blot data, p‐FAK‐Y^397^staining intensity was significantly higher in both USC tumor samples (USC tumor samples: 99.58 ± 11.14 *vs*. EEC tumor samples: 65.14 ± 7.87; *P* = 0.03) and aspirates (USC tumor aspirates: 156.66 ± 11.93 *vs*. EEC tumor aspirates: 114.75 ± 7.51; *P* = 0.006), compared to their EEC counterparts (Fig. 2E). Representative images of p‐FAK immunohistochemical staining confirm higher p‐FAK‐Y^397^ phosphorylation in USC *vs*. EEC (Fig. 2F). Collectively, these results show that FAK kinase presents higher activation in USC compared to EEC as shown by increased Y^397^ phosphorylation and validate kinome profiling approach as an accurate strategy to identify tyrosine kinase activation patterns in cancer.

### 3.5 FAK kinase controls cell growth, proliferation, and migration of USC

**Fig. 3:**
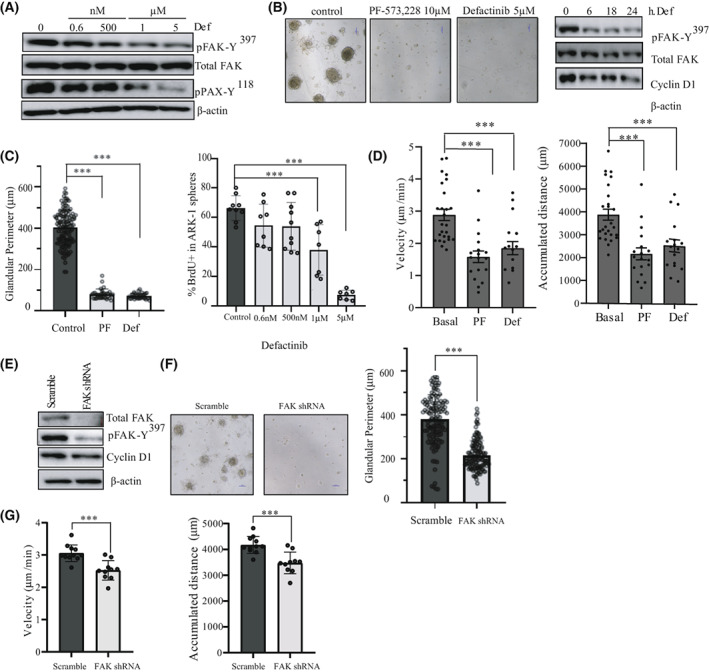
FAK activation controls cell growth and migration in USC cell lines. (A) Representative western blot of p‐FAK‐Y^397^, total FAK, and pPAX‐Y^118^ levels in ARK‐1 cells treated with 0.6 nm, 500 nm, 1 μm, and 5 μm of defactinib for 1 h (*n* = 3 independent experiments). β‐actin expression is used as a loading control. (B) Representative phase contrast images of ARK‐1 3D cells treated with 10 μm PF‐573228 or 5 μm defactinib for 48 h (*n* = 3 independent experiments). Magnification 10×. Scale bar: 100 μm. Representative western blot of p‐FAK‐Y^397^, total FAK, and cyclin D1 levels in ARK‐1 cells treated with 5 μm of defactinib for 0, 6, 18, and 24 h. (*n* = 3 independent experiments). β‐actin expression is used as a loading control. (C) Quantification of glandular perimeter (μm) in ARK‐1 3D cells treated with 10 μm PF‐573228 or 5 μm defactinib for 48 h. Percentage of positive BrdU ARK‐1 cells in a 3D sphere experiment where cells were treated with 0.6 nm, 500 nm, 1 μm, and 5 μm of defactinib for 48 h. Values are means±SD (*n* = 3 independent experiments); One‐way ANOVA test was used followed by Tukey *post hoc* test (***P < 0.001). (D) Quantitative analysis of velocity plot (μm·min^−1^) and accumulated distance (μm) of ARK‐1 cells, treated with either 10 μm PF‐573228 or 5 μm Defactinib. Values are means ± SD (*n* = 3 independent experiments); One‐way ANOVA test was used followed by Tukey *post hoc* test (***P < 0.001). (E) Western blot of p‐FAK‐Y^397^, total FAK and cyclin D1 levels in ARK‐1 scramble and ARK‐1 FAK shRNA cells (*n* = 3 independent experiments). β‐actin expression is used as a loading control. (F) Representative images and quantification of glandular perimeter (μm) in scramble and FAK shRNA ARK1‐3D spheroids (*n* = 3 independent experiments); Student's *t*‐test (***P < 0.001). Scale bar: 100 μm. (G) Quantitative analysis of velocity plot (μm·min^−1^) and accumulated distance (μm) of ARK‐1 scramble *vs*. FAK shRNA cells. Values are means±SD (*n* = 3 independent experiments); Student's *t*‐test (***P < 0.001).

We next wanted to investigate to which extent FAK kinase controls USC tumoral behavior. We treated ARK‐1 USC cell line with increasing doses of defactinib or PF‐573228 for 1 h. Defactinib was able to reduce p‐FAK‐Y^397^ levels at doses ranging from 500 nm to 5 μm with a significant reduction of pPAX‐Y^118^ levels at doses of 1 and 5 μm (Fig. 3A). As for PF‐573228 treated ARK‐1 cells, a significant reduction of p‐FAK‐Y^397^ levels was observed at 10 μm doses (Fig. S4A). Next, we assessed the effect of FAK inhibitors, in a 3D cell model. Exposure of ARK‐1 3D cells to either inhibitor, elicits a significant reduction in sphere size, together with an inhibition of FAK activity and a decrease in Cyclin D1 levels (Fig. 3B). The mean sphere perimeter is 401.7 ± 85.12 μm for control cells *vs*. 80.26 ± 24.32 μm (*P* < 0.0001) for Defactinib‐treated cells and 70.55 ± 12.95 μm (*P* < 0.0001) for PF‐573228‐treated cells (Fig. 3C). This effect on spheroid size is dose‐dependent (Fig. S4B) and is consistent with an inhibitory effect on ARK‐1 spheroid proliferation, reaching statistical significance at the doses of 1 and 5 μm of defactinib (mean of proliferating cells: 66.37 ± 8.45% for control condition *vs*. 37.98 ± 17.11% (*P* = 0.0005) and 7.37 ± 3.24% (*P* < 0.0001) for defactinib 1 μm and defactinib 5 μm‐treated spheroids, respectively; Fig. 3C). This result is in accordance with the pronounced decrease in Cyclin D1 levels in ARK‐1 cells treated with 5 μm defactinib (Fig. 3B). Because of USC's notorious propensity for spreading, we also checked the efficacy of both inhibitors in controlling cell migration. As one of the major drawbacks of collective cell migration assay is its inability to distinguish the effects of anticancer drugs effects on proliferation from migration and based on the anti‐proliferative effects of FAK inhibitors cited above, we next assessed USC cancer migration using time‐lapse imaging tracking individual cells (Videos [Link], [Link]). Cells were seeded at a very low density and tracked using a 2D video microscope device. The quantitative analysis of individual USC cells shows that FAK inhibitors reduce both the migration velocity and the accumulated distance of ARK‐1 cells (Fig. 3D). PF‐573228 and Defactinib‐treated ARK‐1 cells migrated with a reduced velocity, with median values of 1.58 ± 0.18 μm·min^−1^ (*P* < 0.0001) and 1.85 ± 0.21 μm·min^−1^ (*P* = 0.0007), respectively, *vs*. 2.88 ± 0.18 μm·min^−1^ for the control DMSO‐treated counterparts. This result is in accordance with the accumulated distance (cells median accumulated distance value: 2162.22 ± 262.59 μm for PF‐573 228‐treated cells (*P* < 0.0001) and 2544.71 ± 274.09 μm for Defactinib‐treated cells (*P* = 0.001) *vs*. 3877.24 ± 240.01 μm for control cells (Fig. 3D)). We next performed lentivirus‐mediated FAK shRNA depletion (Fig. 3E)]. When allowed to form spheres, ARK‐1 FAK shRNA spheroids displayed a significant decrease of 43% in their perimeter compared to control scr (pLKO) counterparts (mean perimeter for scr: 378.9 ± 112 μm *vs*. 215.8 ± 66.21 μm for FAK shRNA spheroids; *P* < 0.0001) (Fig. 3F) in line with a decrease in Cyclin D1 levels (Fig. 3E). Moreover, FAK shRNA‐transduced cells presented significantly reduced cell velocity compared to their scrambled (pLKO) counterparts [scr: 3.05 ± 0.25 μm·min^−1^; FAK shRNA‐transduced ARK‐1 cells: 2.52 ± 0.30 μm·min^−1^ (*P* = 0.0003)] and accumulated distance [scr‐ARK‐1 cells: 4170 ± 330 μm *vs*. 3474 ± 417.8 μm for FAK shRNA‐transduced cells (*P* = 0.0004); Fig. 3G]. Altogether, these findings demonstrate that FAK controls proliferation, growth, and migration of USC.

### 3.6 FAK activation in USC is linked to oxidative stress

**Fig. 4:**
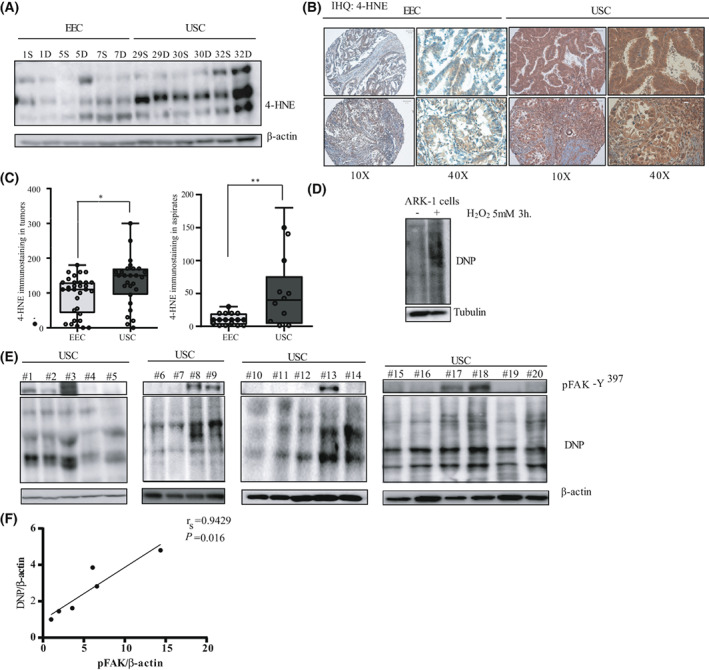
Enhanced oxidative stress markers in USC. (A) Representative western blot of oxidative stress marker 4‐HNE in 12 Endometrial Cancer samples (6 EEC and 6 USC). (*n* = 3 independent experiments). Β‐actin expression is used as a loading control. (B) Representative pictures of 4‐HNE immunostaining in two EEC samples and two USC samples. Pictures were taken at 10× and 40× magnifications. Scale bar: 100 μm. (C) 4‐HNE staining intensity score (immunohistochemistry) was calculated by a histoscore method (being 300 points the maximum immunoreactivity). Values are median ± CI (*n* = 3 independent experiments), statistical analysis was performed to compare 4‐HNE staining intensity in EEC and USC tumor samples and tumor aspirates. Statistical analysis of the obtained results shows a significant increase in 4‐HNE expression in USC tumors compared to EEC ones (Mann–Whitney *U*‐test; *P = 0.03) and in USC tumor aspirates compared to their EEC counterparts (Mann–Whitney *U*‐test; **P = 0.01). (D) Protein carbonylation was analyzed by western blot with anti‐DNP antibody in ARK‐1 cells that were either left untreated or stimulated with 5 mm of H_2_O_2_ for 3 h (*n* = 3 independent experiments). (E) Western blot with anti‐DNP in 20 USC samples (that were previously used for the kinome analysis; *n* = 3 independent experiments). (F) Spearman's rank correlation coefficient analysis comparing p‐FAK‐Y^397^/β‐actin *vs*. DNP/β‐actin protein levels in the six samples (numbers 15–20) of the western blot (Spearman *r*_*s*_ = 0.94; *P* = 0.016).

FAK kinase has a central role as a mediator coordinating several signaling pathways that promote cancer growth and metastasis [17]. FAK overexpression in tumors is often linked to increased FAK mRNA levels and frequently associated with *PTK2* amplification. In endometrial cancer, several groups have reported increased levels of total FAK and p‐FAK‐Y^397^ levels [33, 34]. However, in the analysis performed by the c‐Bioportal oncoprint summary based on the TCGA database, only 8.5% of the total samples presented an altered *PTK2* profile (copy number alteration or mutation) [3, 35, 36]. Recent reports have demonstrated that redox agents and ROS (reactive oxygen species) activate SRC/FAK pathway, which acts as redox sensor to integrate cell survival and metastasis [37]. Under oxidative stress, superoxide anion induces lipid peroxidation of cell membranes 6‐polyunsaturated fatty acids, leading to their oxidative degradation and to the formation of the highly reactive aldehyde 4‐Hydroxy‐2‐nonenal (4‐HNE), which is considered a biomarker of oxidative stress [38]. This aldehyde can then form adducts with cellular proteins, which often results in their dysfunction [38]. We thus assessed the abundance of 4‐HNE‐modified proteins by western blot in samples used for the kinome assay. As shown in Fig. 4A, USC samples presented higher levels of 4‐HNE compared to EEC ones. Moreover, 4‐HNE staining intensity was assessed by immunohistochemistry in an independent panel of 32 EEC and 27 USC samples. Two representative USC and EEC tumor samples stained for 4‐HNE are shown in Fig. 4B and highlight a higher 4‐HNE intensity staining in USC *vs*. EEC samples and aspirates (USC tumor samples: 131.11 ± 13.31 *vs*. EEC tumor samples: 94.22 ± 9.68; *P* = 0.03; USC tumor aspirates: 52.11 ± 15.99 *vs*. EEC tumor aspirates: 10.59 ± 2.14; *P* = 0.01) (Fig. 4C). The clear increase in 4‐HNE in USC *vs*. EEC samples, prompted us to check for protein oxidation in these samples. Protein carbonyl formation is one of the most studied markers under oxidative stress conditions [39]. By way of control, we first stimulated ARK‐1 cells with H_2_O_2_ for 24 h. As expected, H_2_O_2_‐stimulated cells displayed an increase in protein carbonylation compared to the control condition (Fig. 4D). Next, 20 tumor samples used for the kinome analysis were derivatized with DNPH and subjected to SDS/PAGE electrophoresis followed by western blot with an anti‐DNP antibody. As shown in Fig. 4E, USC samples displayed higher levels of protein damage compared to EEC samples. We next checked whether there was any correlation between DNP levels and p‐FAK Y^397^ activation levels in USC samples. As shown in Fig. 4F, a positive correlation between p‐FAK‐Y^397^ and DNP levels was observed when lysates from the same western blot (samples 15–20) were compared (Spearman test, *r*_*s*_ = 0.94; *P* = 0.016). Altogether, these results strongly suggest that oxidative stress is increased in USC, and that ROS are potential mediators of FAK activation.

### 3.7 Oxidative stress activates FAK and promotes single‐cell migration in USC cell line

**Fig. 5:**
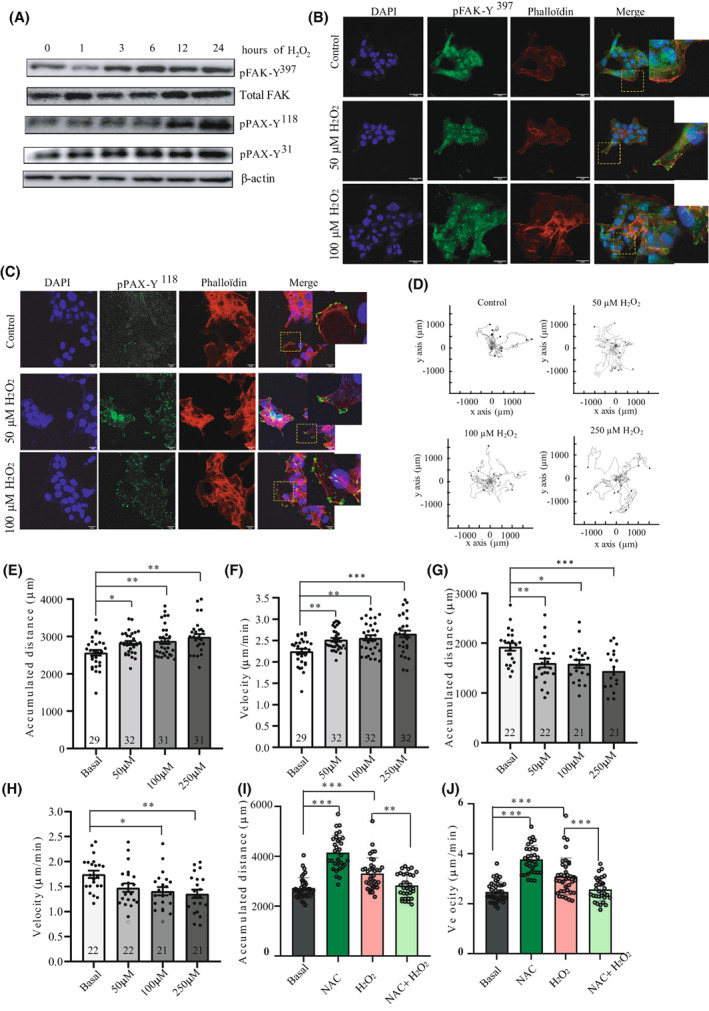
FAK activation under oxidative stress enhances USC single‐cell migration and directionality. (A) Representative western blot of ARK‐1 cells stimulated with 0.5 mm of H_2_O_2_ at the indicated times (*n* = 3 independent experiments). Cell lysates were immunoblotted with antibodies against p‐FAK‐Y^397^, total FAK, p‐PAX‐Y^31^, p‐PAX‐Y^118^. Β‐actin was used as a loading control. (B) Representative images of immunofluorescence staining of p‐FAK‐Y^397^ in control and H_2_O_2_‐stimulated (50 and 100 μm) ARK‐1 cells, assessed by confocal microscopy (*n* = 3 independent experiments). DAPI (blue), p‐FAK‐Y^397^ (green), and phalloidin (red). Scale bar: 20 μm. (C) Representative images of immunofluorescence staining of p‐PAX (Y^118^) in control and H_2_O_2_‐stimulated (50 and 100 μm) ARK‐1 cells, assessed by confocal microscopy DAPI (blue), p‐PAX‐Y^118^ (green), and phalloidin (red) (*n* = 3 independent experiments). Scale bar: 20 μm. (D) Trajectory plots showing control and H_2_O_2_‐stimulated (50, 100, and 250 μm) ARK‐1 cells trajectory for 20 h (*n* = 3 independent experiments). All tracks were set to a common origin (intersection of x and y axes) using Chemotaxis plugin of ImageJ/Fiji. (E) Quantitative analysis of accumulated distance (μm) and (F) velocity (μm·h^−1^) of single ARK‐1 control cells and cells treated with 50, 100, and 250 μm H_2_O_2_. Values are mean ± SD (*n* = 3 independent experiments); One‐way ANOVA was used followed by Tukey *post hoc* test (*P < 0.05; **P < 0.01; ***P < 0.001). (G) Quantitative analysis of accumulated distance (μm) and (H) velocity (μm·h^−1^) of single ARK‐1 control cells and cells treated with 50, 100, and 250 μm H_2_O_2_ and 5 μm Defactinib. Values are mean ± SD (*n* = 3 independent experiments); One‐way ANOVA was used followed by Tukey *post hoc* test (*P < 0.05; **P < 0.01; ***P < 0.001). (I) Quantitative analysis of accumulated distance (μm) and (J) velocity (μm·h^−1^) of single ARK‐1 control cells and cells treated with 1 mm NAC, 250 μm H_2_O_2_, and 1 mm NAC + 250 μm H_2_O_2_. Values are mean ± SD (*n* = 3 independent experiments); One‐way ANOVA was used followed by Tukey *post hoc* test (**P < 0.01; ***P < 0.001).

Since recent studies have shown that ROS signaling controls cell migration in a breast cancer cell model [40], we set to assess the effects of oxidative stress on migration of USC cells. We first stimulated the USC ARK‐1 cells with H_2_O_2_. As shown in Fig. 5A, H_2_O_2_ stimulation induces a sustained activation of p‐FAK‐Y^397^ starting 3 h' post‐stimulation. Of note, this activation is maintained for the 24 h of stimulation. For efficient cell migration the adhesion contacts need to assemble and disassemble [41]. One of the best‐characterized substrates of FAK‐mediated phosphorylation is Paxillin. As shown in Fig. 5A, ARK‐1 cells treated with H_2_O_2_ increased Paxillin‐Y^118^ and Paxillin‐Y^31^ phosphorylation levels. We next performed immunofluorescence analysis to check for p‐FAK‐Y^397^ and pPAX‐Y^118^ subcellular localization. As shown in Fig. 5B, while p‐FAK‐Y^397^ has a predominantly cytoplasmic localization in untreated ARK‐1 cells, it localized to the FA when cells were stimulated with H_2_O_2_. Moreover, pPAX‐Y^118^ staining intensity in the FA was higher in H_2_O_2_‐treated cells compared to control cells (Fig. 5C). Next, we hypothesized whether the oxidative stress could affect cell migration. To answer this question, we performed 2D video microscopy of cells seeded at low density and tracked cell movement (Videos [Link], [Link]). Cells were either left untreated or were stimulated with increasing doses of H_2_O_2_ for 24 h. Cell tracking analyses show that H_2_O_2_‐treated cells present an increased directionality pattern (Fig. 5D). Specifically, the quantitative analysis shows that H_2_O_2_‐stimulated ARK‐1 cells migrated with a significant increased velocity [control condition: 2.24 ± 0.32 μm·min^−1^; 50 μm H_2_O_2_‐treated cells: 2.52 ± 0.25 μm·min^−1^ (*P* = 0.01); 100 μm H_2_O_2_‐treated cells: 2.56 ± 0.35 μm·min^−1^ (*P* = 0.01) and 250 μm H_2_O_2_‐treated cells: 2.65 ± 0.44 μm·min^−1^ (*P* < 0.0001)] and showed an increase in total accumulated distance [control condition: 2564 ± 75.78 μm; 50 μm H_2_O_2_‐treated cells: 2825 ± 52.23 μm (*P* = 0.05); 100 μm H_2_O_2_‐treated cells: 2874 ± 75.05 μm (*P* = 0.01) and 250 μm H_2_O_2_‐treated cells: 2985 ± 78.81 μm (*P* = 0.0004); Fig. 5E,F]. Next, in order to check whether H_2_O_2_‐induced migration is FAK dependent, we decided to treat the cells with the FAK inhibitor defactinib (VS‐6063). We thus treated H_2_O_2_‐stimulated ARK‐1 cells with 5 μm Defactinib. As shown in Fig. 5G,H, the increased migration observed in H_2_O_2_‐treated ARK‐1 cells was completely lost when cells were also treated with FAK inhibitor defactinib. H_2_O_2_‐treated ARK‐1 cells stimulated with 5 μm defactinib show a significant reduction in velocity [control condition: 1.75 ± 0.33 μm·min^−1^; 50 μm H_2_O_2_ treated cells: 1.48 ± 0.40 μm·min^−1^ (*P* = n.s.); 100 μm H_2_O_2_‐treated cells: 1.41 ± 0.38 μm·min^−1^ (*P* = 0.02) and 250 μm H_2_O_2_‐treated cells: 1.35 ± 0.38 μm·min^−1^ (*P* = 0.005)] and in accumulated distance [control condition: 1925 ± 76.30 μm; 50 μm H_2_O_2_‐treated cells: 1599 ± 88.22 μm (*P* = 0.01); 100 μm H_2_O_2_‐treated cells: 1584 ± 79.44 μm (*P* = 0.02) and 250 μm H_2_O_2_‐treated cells: 1440 ± 88.19 μm (*P* = 0.0005); Fig. 5G,H]. Next, we checked the role of ROS scavenger N‐Acetylcysteine (NAC) on cell migration. As shown in Fig. 5I,J, NAC alone increased ARK‐1 cell migration, with increased cell velocity parameter [control: 2.47 ± 0.39 μm·min^−1^; 1 mm NAC‐treated cells: 3.77 ± 0.56 μm·min^−1^ (*P* < 0.0001)] and accumulated distance [control condition: 2727 ± 417.6 μm; 1 mm NAC‐treated cells: 3.77 ± 0.56 μm·min^−1^ (*P* < 0.0001)] and accumulated distance [control condition: 2727 ± 417.6 μm; 1 mm NAC‐treated cells: 4160 ± 682.7 μm (*P* < 0.0001)]. Interestingly, the effect of H_2_O_2_ on cell migration was almost completely reversed by NAC. NAC efficiently abolished the velocity increase observed in H_2_O_2_‐treated cells [250 μm H_2_O_2_‐treated cells: 3.09 ± 0.74 μm·min^−1^ and 1 mm NAC + 250 μm H_2_O_2_‐treated cells 2.56 ± 0.46 μm·min^−1^ (*P* = 0.00008); Fig. 5I] and the increased accumulated distance [H_2_O_2_‐treated cells: 3315 ± 628.6 μm; 1 mm NAC + 250 μm H_2_O_2_‐treated cells: 2821 ± 476.1 μm (*P* = 0.002); Fig. 5J]. Taken together, these results point out at FAK as a mediator of oxidative‐stress‐induced migration in USC cells and confirm the role of ROS as important drivers of this migration.

### 3.8 FAK inhibition reduces USC cell growth in CTOS and PDOX *in vivo* model

**Fig. 6:**
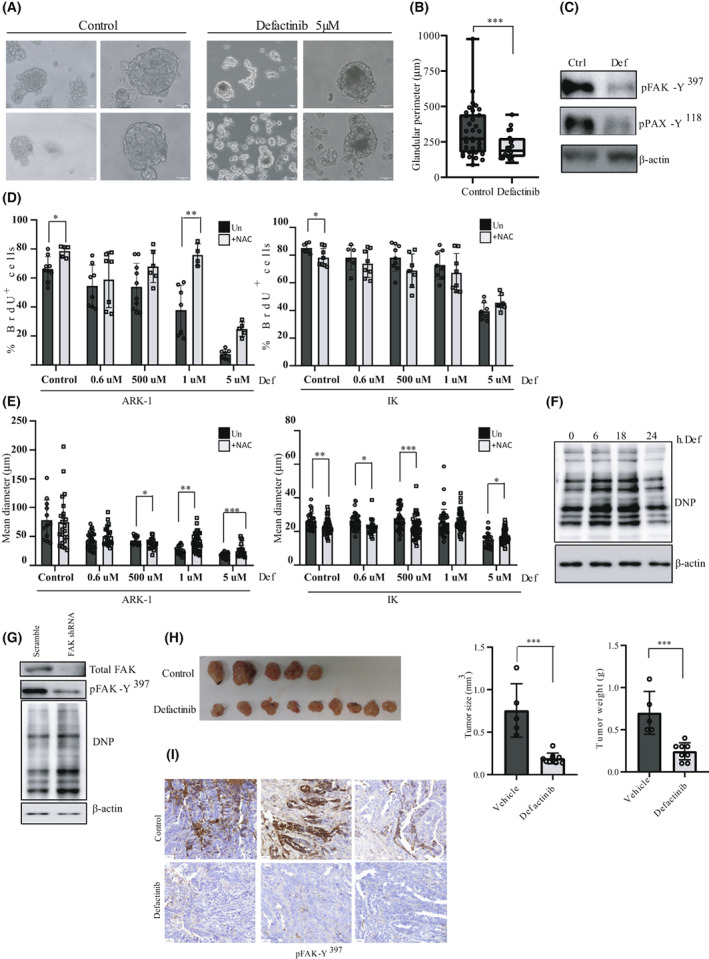
FAK inhibition reduces CTOS model cell viability and *in vivo* PDOX tumor growth of USC. (A) Representative phase contrast images of CTOS in untreated (control) and treated (5 μm Defactinib) cells for 24 h (*n* = 3 independent experiments). A magnification is shown, Scale bar: 30 μm. (B) Quantitative analysis of CTOS glandular perimeter (μm) in untreated and Defactinib‐treated spheroids. Values are median ± CI (*n* = 3 independent experiments; Mann–Whitney *U*‐test; ***P = 0.004). (C) Representative western blot of CTOS in untreated and Defactinib‐treated cells. Cell lysates were immunoblotted with antibodies against p‐FAK‐Y^397^ and p‐PAX‐Y^118^ (*n* = 3 independent experiments). Β‐actin was used as a loading control. (D) Percentage of BrdU‐positive cells in ARK‐1 and IK spheroid cultures. Cells were either left untreated or were pre‐treated with 1 mm NAC before stimulation at the indicated doses of Defactinib, and then allowed to form 3D spheroid cultures for 48 h, after which BrdU was added to the spheroid cultures. Values are mean ± SD (*n* = 3 independent experiments); Student's *t*‐test (*P < 0.05; **P < 0.01). (E) Quantification of spheroid mean diameter (μm) in 3D spheroids from ARK‐1 and IK cells. Values are mean ± SD (*n* = 3 independent experiments); Student's *t*‐test (*P < 0.05; **P < 0.01; ***P < 0.001). (F) Representative western blot against DNP in ARK‐1 cells stimulated for the indicated times with defactinib 5 μm (*n* = 3 independent experiments). (G) Representative western blot against p‐FAK‐Y^397^, total FAK, and DNP in scramble and lentiviral transduced FAK shRNA cells. β‐actin was used as a loading control (*n* = 3 independent experiments). (H) Images of PDOX tumors at mice sacrifice in untreated (control) and treated (100 mg·kg^−1^ Defactinib) conditions. Quantitative analysis of tumor size (mm^3^) and weight (g) in vehicle and Defactinib‐treated tumors. Values are mean ± SD; Student's *t*‐test (***P = 0.0002 and *P* = 0.0004, respectively). (I) Representative pictures of p‐FAK‐Y397 immunostaining in control and defactinib PDOX tumors. Scale bar: 50 μm.

In the last 10 years, CTOS (cancer‐tissue‐originated spheroids) have emerged as a powerful alternative to conventional 2D cultures, as this model maintains original cell–cell contact of cancer cells greatly mimicking the original human tumor [42]. Moreover, cellular heterogeneity and many histological features of the primary tumor are retained in CTOS rendering them a useful tool for chemosensitivity assays [43]. Importantly, spheroid models may also be of great value in assessing the clinical outcome of individual patients in response to therapy. Thus, USC cancer tissue was removed from a cancer patient as previously described [42] and purified. Sixteen hours later, the spheroid formation was observed. The medium was then changed, and spheroids were either left untreated or treated with defactinib for 24 h. Defactinib inhibited CTOS spheroid growth (Fig. 6A), as shown by a significant decrease in spheroids glandular perimeter [control: 324.2 ± 179.3 μm *vs*. 5 μm Defactinib‐treated spheroids: 215.5 ± 87.75 μm (*P* = 0.004); Fig. 6B]. Cell lysate analysis shows a significant decrease in FAK‐Y^397^ and Paxillin‐Y^118^ levels in Defactinib‐treated CTOS compared to their control counterparts (Fig. 6C). We next wanted to check whether the reduction of spheroid growth in the presence of defactinib depends on p‐FAK‐Y^397^ initial levels. We have therefore performed a 3D experiment with IK and ARK‐1 cells presenting low and high levels of p‐FAK‐Y^397^, respectively. We performed a BrdU incorporation assay and measured the mean diameter of the spheroids formed by ARK‐1 and IK cells. Defactinib reduces much more efficiently both BrdU incorporation and spheroid diameter in USC cell line than in EEC cell line, a result that is in line with the levels of p‐FAK‐Y^397^ (Fig. 6D,E, for BrdU incorporation, and mean spheroid diameter, respectively). As such, while 1 μm of defactinib significantly reduced BrdU incorporation in ARK‐1 cell line by 42.77%, it did not affect the proliferation rate of IK cells. Similar results are also observed when the diameter of the spheroids is assessed. As such, whereas ARK‐1 diameter presented a reduction of 45.27% compared to control condition when treated with the lowest defactinib dose (0.6 nm), IK spheroid diameter was not affected by defactinib doses ranging from 0.6 nm to 1 μm. Altogether these results indicate that FAK inhibitors reduce the proliferation of USC cells and that the extent of BrdU inhibition depends on the levels of p‐FAK‐Y^397^. Next, and to elucidate the role of ROS in the observed phenotype, we pre‐treated the cell cultures with the ROS scavenger NAC (1 mm). Our results show that NAC pretreatment restores the proliferation rate and the mean diameter in ARK‐1 cells but not in IK cells (Fig. 6D,E). One explanation would be that defactinib inhibits ARK‐1 cell proliferation by increasing ROS, and that NAC would restore cell proliferation by relieving oxidative stress. This result indicates that while oxidative stress is an activator of FAK kinase in USC, FAK itself has an antioxidant role. To test this hypothesis, ARK‐1 cells were treated with defactinib at different times, and cell lysates were checked for carbonyl oxidation markers. As shown in Fig. 6F, cells treated with defactinib presented an increase in DNP levels, with ratios of DNP/β‐actin increasing with defactinib treatment (control: DNP/β‐actin = 1, defactinib 6 h: DNP/β‐actin = 1.15; defactinib 18 h: DNP/β‐actin = 1.11 and defactinib 24 h: DNP/β‐actin = 1.38). This result was also confirmed in FAK shRNA transduced cells with a 2.68‐fold increase in the DNP/β‐actin ratio in FAK shRNA cells compared to scr counterparts. Next, we aimed to assess *in vivo* the effects of Defactinib. We first tested defactinib doses in an *in vivo* mouse xenograft model of ARK‐1 cells and confirmed a decrease in FAK activation levels at the dose of 100 mg·kg^−1^·day^−1^ (Fig. S5). After assessing the defactinib dose, we implanted orthotopically in nude mice females a small fragment of primary human USC, as described [30]. This orthoxenograft model presents the advantages of retaining the molecular and histological characteristics of the original tumor and is a great tool for drug testing due to its high predictive drug‐response values [30, 44]. Of note, in the orthoxenograft model, we were unable to appreciate neither a vascular nor myometrial invasion of the cells in the control condition. However, Defactinib‐treated mice presented a significant decrease in tumor size and weight compared to vehicle‐treated counterparts (Fig. 6H), and this decrease was accompanied with a decrease in p‐FAK‐Y397 levels as assessed by immunohistochemistry (Fig. 6I). Altogether these results point to FAK activation pathway as a key antioxidant pathway that controls USC tumorigenesis.

## 4 Discussion

Despite its uncommon occurrence, USC contributes to almost 80% of endometrial cancer–related deaths. This cancer is characterized by extremely high recurrence rates that frequently arise from metastatic relapse. Currently, the most frequent approach for USC treatment is surgery followed by chemotherapy and radiotherapy [45]. It is worth mentioning that women with advanced‐stage or recurrent USC who overexpress epidermal growth factor receptor 2 HER2/neu can obtain a meaningful clinical benefit from a treatment combining trastuzumab, a humanized monoclonal antibody against (HER2)/neu and Carboplatin‐paclitaxel [46]. However, further investigation is needed for the elaboration of new targeted therapies in this cancer, especially for HER2‐negative patients. In recent years, genetic studies have provided a mine of invaluable data regarding genomic profiling of Endometrial Cancer (EC) [3]. However, in the clinic, genetic alterations are difficult to target. In this context, tyrosine kinases constitute potential targets for USC management that may be added to genetic‐based biomarkers, providing thus a more complete approach to tackle this cancer.

In this study, we used high‐throughput technique (Pamgene) to compare tyrosine kinase activity profiles of USC *vs*. EEC. FAK1 appeared as an interesting target since it showed an overall increase in activity (upstream kinase analysis) and an increase in the phosphorylation of a peptide‐containing tyrosine 576 and 577, located in the kinase domain and associated with a full activation of FAK kinase [47]. Interestingly, one of the kinases shown to phosphorylate these two residues, the FER kinase, also shows increased activity in USC *vs*. EEC samples (Figs S1B and S2A) [48]. We validated increased FAK activation in USC tumor samples, by WB and IHC. FAK phosphorylation is triggered by integrin‐mediated cell adhesion. Various studies have demonstrated the role of FAK in tumor angiogenesis, migration, invasion, and metastasis [49, 50, 51] and also as a regulator of different protein complexes that connect the cytoskeleton to the extracellular matrix, controlling thus cell adhesion and motility [11]. FAK overexpression and increased phosphorylation levels have also been associated with high histologic grade, lymph node metastasis, and myometrial invasion in uterine cancer [51] and correlate with worse OS (overall survival) in different types of cancers including endometrial cancer (HR = 4.15, 95% CI: 2.83–6.08, *P* = 0) [51, 52].

In our cell line model, by using wound healing assays and time‐lapse microscopy for single cell tracking, we demonstrate a role for FAK activation in controlling USC migration. In fact, either pharmacological inhibition or shRNA‐lentiviral‐mediated depletion of FAK significantly reduced USC cell migration. Moreover, using the same approach, we also show that FAK controls USC spheroids' cell growth and proliferation rate, and that its inhibition reduces cell proliferation with a concomitant decrease in total cyclin D1 levels.

Reactive oxygen species (ROS) are known to be key intermediaries in many signaling pathways. Our *in vitro* results show an increase in oxidative stress markers in USC *vs*. EEC, such as 4‐HNE or protein carbonyl oxidation marker DNP. Finally, we demonstrated that ROS signaling is implicated in FAK activation in USC and that ROS‐induced activation of the FAK‐Paxillin pathway increases cell migration. Interestingly, increased mitochondrial superoxide production has been shown to promote metastasis via Src activation [53]. These findings are in line with our results as Src is an upstream activator of FAK. Together, these findings suggest that increased ROS levels promote the metastatic behavior of USC by activating FAK signaling, identifying this tyrosine kinase as a potential target for USC management.

In conclusion, we show that aberrant FAK activation promotes tumor migration in USCs, and that oxidative stress acts very likely as a promoter of this activation (Fig. S6). Moreover, FAK activation plays a protective antioxidant role that would aim to protect cells from excessive oxidative damage, promoting their proliferation. This result is in agreement with a growing body of evidences that supports the view that antioxidant activities are essential for tumorigenesis [54]. *In vitro* results were further confirmed *in vivo* by CTOS and PDOX models, where we have shown that defactinib efficiently reduced the activation FAK pathway and tumor growth. In this context and as reproducible results for FAK activation were obtained both in aspirates and tumor specimens, p‐FAK‐Y^397^ shows promise as a potential biomarker to be assessed in tumor specimen or aspirates, to determine tumor aggressiveness and potential responsiveness to FAK kinase inhibition. Moreover, mitochondrial ROS scavengers can also be assessed for metastasis prevention. This approach has been already exploited in human breast cancer model, where mitoTEMPO superoxide scavenger has proven to prevent spontaneous metastasis of these cells in a mouse model, preserving physiological ROS signaling [53]. Taken together, our findings highlight the emergence of FAK kinase as a promising candidate for the management of USC, and strongly suggest that the inhibition of FAK pathway may be of clinical benefit and thus improve USC patient's survival.

## 5 Conclusions

Uterine serous carcinoma (USC) is an aggressive form of endometrial cancer that presents a high degree of resistance to standard therapeutic approaches. Here, we report that kinome profiling of patient‐derived USCs presents a specific signature associated with an increase in FAK kinase activity. ROS appear as a principal driver of this activation. FAK activation has an antioxidant role that promotes cell growth. Complementary pharmacologic and genetic approaches to block FAK activity decreased tumor growth and migration *in vitro*. Moreover, pharmacological inhibition of FAK reduced tumor growth in a PDX model. Our findings illustrate that endometrial cancer kinome profiling may be useful as a prognostic tool. Further investigations are needed to elucidate the interaction between oxidative stress and FAK kinase activation in this type of tumors.

## Conflict of interest

A. Villanueva and A. Vidal are co‐founders of Xenopat S.L. The rest of authors declare no competing interests.

## Author contributions

AY, XMG, and EG designed the study. AY wrote the draft. ILM and JP performed data analysis and generated figures and tables together and revised the manuscript. ME, EC, and LF provided guidance and supervision. SG, Ana Velasco, and MP provided the patient samples and classified tumor samples. GC and JP and RN performed data and statistical analysis. AY and ILM performed kinome experiments. AY performed the western blots and confocal microscopy experiments. MS performed the immunohistochemistry staining and AP performed the cell tracking assays and analysis. LC, LPP, NB, and DLN performed the CTOS (cancer‐tissue‐originated spheroids) experiments and analysis while August Vidal and Alberto Villanueva performed the PDOX *in vivo* model. All authors read and approved the final version of manuscript.

### Peer review

The peer review history for this article is available at https://publons.com/publon/10.1002/1878‐0261.13346.

## Supporting information


**Fig. S1.** Overall survival analysis after surgery.
**Fig. S2.** Volcano plot showing the putative kinases differentially activated between EEC and USC tumor samples, with their final score (Q) and specificity score.
**Fig. S3.** pFAK‐Y^397^ antibody specificity controls.
**Fig. S4.** FAK activation controls USC cell line growth.
**Fig. S5.** Defactinib dose testing in ARK‐1 xenograft model.
**Fig. S6.** Schematic depicting the role of oxidative stress in activating FAK signaling pathway in USC.
**Table S1.** Histological, clinical, and molecular classification of the cases.

**Video S1.** Representative single cell‐tracking video in control ARK‐1 cells.

**Video S2.** Representative single cell‐tracking video in 5 μM Defactinib‐ARK‐1‐treated cells.

**Video S3.** Representative single cell‐tracking video in 10 μM PF‐573228 ARK‐1‐treated cells.

**Video S4.** Representative single cell‐tracking video in control ARK‐1 cells.

**Video S5.** Representative single cell‐tracking video in ARK‐1 cells treated with 50 μM H_2_O_2_.

**Video S6.** Representative single cell‐tracking video in ARK‐1 cells treated with 100 μM H_2_O_2_.

## Data Availability

Data supporting these findings are reported in the Supplementary Information. Information related to the results of this study is available from the corresponding author (andree.yeramian@udl.cat) upon request.
