# Supplementary material for: Oxidative stress‐induced FAK activation contributes to uterine serous carcinoma aggressiveness
Source: Mol Oncol. 2022 Dec 7;17(1):98–118. doi: 10.1002/1878-0261.13346 (PMC9812840; doi:10.1002/1878-0261.13346)
Supplement: Supplementary file 1 — Fig. S1. Overall survival analysis after surgery. Fig. S2. Volcano plot showing the putative kinases differentially activated between EEC and USC tumor samples, with their final score (Q) and specificity score. Fig. S3. pFAK‐Y397 antibody specificity controls. Fig. S4. FAK activation controls USC cell line growth. Fig. S5. Defactinib dose testing in ARK‐1 xenograft model. Fig. S6. Schematic depicting the role of oxidative stress in activating FAK signaling pathway in USC. Table S1. Histological, clinical, and molecular classification of the cases. [file MOL2-17-98-s006.docx]

**Oxidative-stress-induced FAK activation contributes to Uterine Serous Carcinoma (USC) aggressiveness**

Isabel C. Lopez-Mejia*, Jordi Pijuan*, Raúl Navaridas, Maria Santacana, Sònia Gatius, Ana Velasco, Gerard Castellà, Anaïs Panosa, Elisa Cabiscol, Miquel Pinyol, Laura Coll, Núria Bonifaci, Laura Plata Peña, August Vidal, Alberto Villanueva, Eloi Gari, David Llobet-Navàs, Lluis Fajas, Xavier Matias-Guiu^$^, Andrée Yeramian^$^

* These authors contributed equally to this work

^$^ These authors are senior co-authors

**Supporting information**

**Supplementary Figures Page**

**Fig. S1**  3

**Fig. S2** 4

**Fig. S3** 5

**Fig. S4** 6

**Fig. S5** 7

**Fig. S6** 8

**Table S1** 9-10

**Supplementary Videos**  11

**Supplementary Figure S1**

**Supplementary Figure S1. Overall survival analysis after surgery.** Survival curves of patients with EEC and USC (for details see **Supplementary Table S1**) shows high levels of significance between both groups, with USC group presenting a shorter overall survival (Gehan-Breslow generalized Wilcoxon test, *p*=0.0018). Both groups of patients were followed-up for a period of 7 years from February 2014 till February 2021.

**Supplementary Figure S2**

**A.**


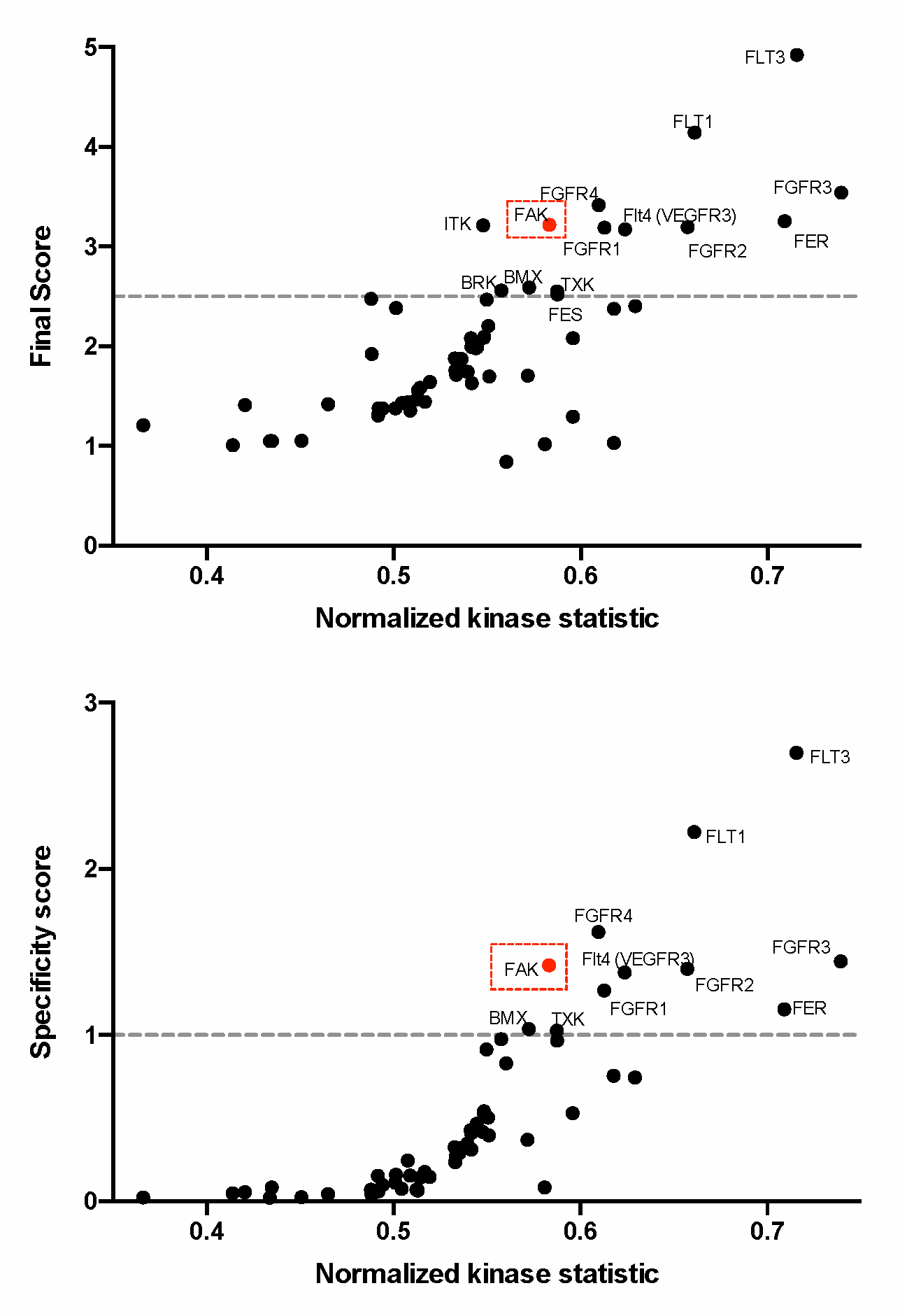


**B.**

**Supplementary Figure S2. Volcano plot showing the putative kinases differentially activated between EEC and USC tumor samples, with their final score (Q) and specificity score. A.** Volcano plots representing the final score of putative upstream tyrosine kinase activity profiling ranked by their final score and **B.** specificity score. The x-axis indicates the value of the kinase statistic. A positive value of the kinase statistic means that kinase activity is higher in the USC group compared to the EEC group. Only kinases with a final score higher than 2.5 are labelled in the graph A.

**Supplementary Figure S3**

**A.**

scr

FAK shRNA

**B.**

**Supplementary Figure S3. A. pFAK-Y^397^ antibody specificity controls.** ARK-1 cells were transduced either with pLKO scr lentiviral vector, or the vector harbouring FAK shRNA. Puromycin resistant cells were selected and pFAK-Y^397^ was assessed by Western Blot. Cells were then collected and mixed in 3% of agar, and fixed with formol. pFAK-Y^397^ staining was performed using the pFAK-Y^397^ as the same concentration used for tumors staining**. B.** Secondary antibody control. USC samples were probed with secondary antibody with no primary antibody.

**Supplementary Figure S4**

**A.**

nM

µM

0 4 500 1 10 PF-573,228

Total FAK

pFAK-Y^397^

β-actin

**B.**

Def 0.6 nM

Def 500 nM

Def 1 uM

Def 5 uM

Control

**
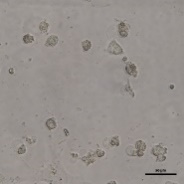

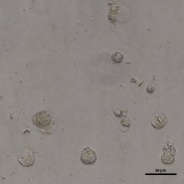

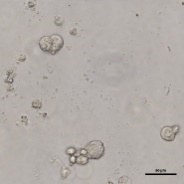

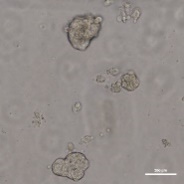

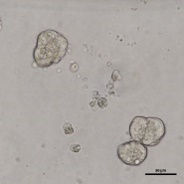
**

**ARK-1**

**Supplementary Figure S4. FAK activation controls USC cell line growth.**

**A.** ARK-1 cells treated with increasing doses of PF-573,228 for 1 hour, and protein lysates blotted for pFAK-Y^397^, total FAK and β-actin. **B.** Defactinib doses-dependent reduction of cell growth: Phase contrast microscopy pictures of 3D ARK-1 spheroid cultures treated with increasing doses of Defactinib.

**Supplementary Figure S5**

**A.**

0 3 6 12 hours Defactinib (100 mg/kg)


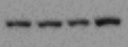
 130KDa pFAK-Y^397^


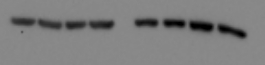
 Total FAK

β-actin

**B.**

 ****

**Vehicle Defactinib (100 mg/kg)**

**Supplementary Figure S5. A. Defactinib dose testing in ARK-1 xenograft model**: pFAK-Y^397^ in ARK-1 xenografts, in mice treated with defactinib at the dose of 100 mg/kg/animal/day. Samples were surgically removed 3, 6 and 12 hours post-defactinib treatment, and analyzed by Western blot. **B.** IHC staining of PDX USC samples, showing the hole surgically removed specimen. Scale bar: 1000 µm.

**Supplementary Figure S6**

**
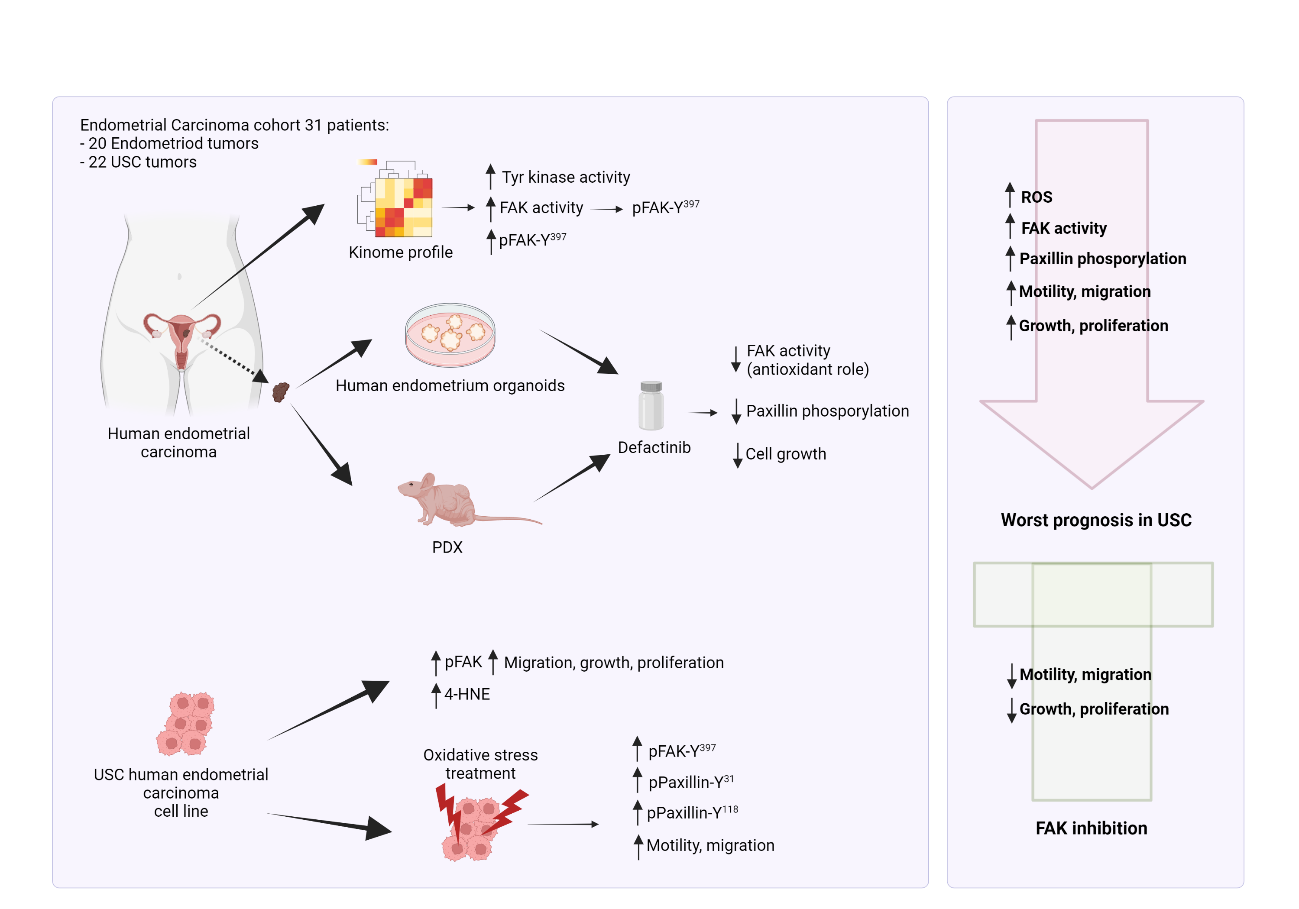
**

**Supplementary Figure S6. Schematic depicting the role of oxidative stress in activating FAK signaling pathway in USC.** The pharmacological inhibition of FAK activity reduces motility and growth, rendering FAK inhibitors promising drugs to tackle USC and improve patient’s survival.

EEC (Endometrial Endometrioid Carcinoma); USC (Uterine Serous Carcinoma); POLε (POLε mutated case); MSI (microsatellite instability, hiper-mutated case); LCN (low copy number); HCN (high copy number); NA (not assessed); DOD (Dead of Disease); NED (No evidence of disease; DAD (Death of another disease).

| ID | AGE AT DIAGNOSIS (years) | DATE OF SURGERY | HISTOLOGICAL TYPE | GRADE | STAGE | MOLECULAR CLASIFICATION | RECURRENCE/METASTASIS | FOLLOW UP | LAST DATE FOLLOW UP | | DATE OF DEATH |
| --- | --- | --- | --- | --- | --- | --- | --- | --- | --- | --- | --- |
| T1 | 79 | 18/02/2014 | EEC | II | pT3a | MSI | YES | DOD | 14/04/2014 | 30/04/2014 | |
| T2 | 78 | 04/03/2014 | EEC | I | pT1a | LCN | NO | NED | 28/19/2020 |  | |
| T4 | 56 | 12/03/2014 | EEC | II | pT1a | MSI | NO | NED | 02/02/2021 |  | |
| T5 | 69 | 09/04/2014 | EEC | II | pT1a | MSI | NO | NED | 21/06/2020 |  | |
| T6 | 85 | 23/04/2014 | EEC | II | pT1a | LCN | NO | NED | 05/11/2019 |  | |
| T7 | 79 | 17/07/2014 | EEC | II | pT1b | LCN | NO | NED | 25/06/2020 |  | |
| T8 | 76 | 17/07/2014 | EEC | II | pT1a | LCN | NO | NED | 09/10/2020 |  | |
| T9 | 60 | 23/07/2014 | EEC | I | pT1b | LCN | NO | NED | 16/11/2020 |  | |
| T10 | 65 | 28/07/2014 | EEC | II | pT3c | LCN | YES | DOD | 28/06/2017 | 28/06/2017 | |
| T11 | 84 | 30/07/2014 | EEC | III | pT1b | LCN | NO | NED | 09/06/2020 |  | |
| T12 | 71 | 05/08/2014 | EEC | III | pT1b | POLε | NO | DAD | 01/07/2016 | 01/07/2016 | |
| T13 | 64 | 26/08/2014 | EEC | I | pT1a | LCN | NO | NED | 22/12/2020 |  | |
| T14 | 85 | 28/08/2014 | EEC | II | pT1b | MSI | NO | DAD | 26/08/2019 | 26/08/2019 | |
| T15 | 86 | 23/09/2014 | EEC | I | pT1b | MSI | NO | NED | 27/08/2020 |  | |
| T16 | 80 | 31/10/2014 | EEC | II | pT1b | MSI | NO | DAD | 02/03/2017 | 02/03/2017 | |
| T17 | 70 | 13/11/2014 | EEC | I | pT1a | MSI | NO | NED | 07/11/2019 |  | |
| T18 | 45 | 24/02/2015 | EEC | II | pT1a | POLε | NO | NED | 02/03/2021 |  | |
| T19 | 39 | 12/02/2014 | EEC | II | pT3c | LCN | NO | NED | 28/10/2020 |  | |
| T20 | 86 | 11/02/2014 | EEC | I | pT1a | LCN | NO | NED | 27/07/2018 |  | |
| T21 | 64 | 13/01/2014 | EEC | II | pT1b | MSI | NO | NED | 30/06/2020 |  | |
| T22 | 81 | 05/05/2010 | USC | III | pT3a | HCN | YES | DOD | 20/08/2014 | 20/08/2014 | |
| T23 | 91 | 13/12/2013 | USC | III | pT2 | HCN | NO | DAD | 07/06/2016 | 07/06/2016 | |
| T24 | 76 | 04/06/2014 | USC | III | pT1a | HCN | YES | DOD | 09/07/2016 | 09/07/2016 | |
| T25 | 81 | 02/07/2014 | USC | III | pT3b | HCN | YES | DOD | 01/01/2016 | 01/01/2016 | |
| T26 | 58 | 04/12/2014 | USC | III | pT3c | LCN | YES | DOD | 31/03/2017 | 31/03/2017 | |
| T27 | 83 | 23/12/2014 | USC | III | pT3a | HCN | YES | DOD | 01/07/2016 | 01/07/2016 | |
| T28 | 74 | 11/02/2015 | USC | III | pT3a | HCN | YES | DOD | 13/10/2018 | 13/10/2018 | |
| T29 | NA | NA | USC | III | NA | HCN | NA | NA | NA |  | |
| T30 | NA | NA | USC | III | NA | HCN | NA | NA | NA |  | |
| T31 | NA | NA | USC | III | NA | HCN | NA | NA | NA |  | |
| T32 | 76 | 16/11/2006 | USC | III | pT3b | HCN | NA | NA | NA |  | |

**Supplementary Table S1.** Histological, clinical and molecular classification of the cases.

**Supplementary Table S1.** EC tumors have been classified into four categories: 1) Ultra-mutated with pathogenic POLε mutations; 2) Hyper-mutated with altered immunoexpression of MMR (DNA mismatch repair) proteins; 3) High copy number, with mutated immunoexpresion pattern of p53 and 4) Low copy number, with normal MMR proteins expression, wild-type p53 expression and no mutations in POLε. Both groups of patients were followed-up for a period of 7 years from February 2014 till February 2021.

**Supplementary Videos**

**Video S1.** Representative single cell-tracking video in control ARK-1 cells.

**Video S2.** Representative single cell-tracking video in 5 µM Defactinib-ARK-1 treated cells.

**Video S3.** Representative single cell-tracking video in 10 µM PF-573,228 ARK1-treated cells.

**Video S4.** Representative single cell-tracking video in control ARK-1 cells.

**Video S5.** Representative single cell-tracking video in ARK-1 cells treated with 50 µM H_2_O_2_.

**Video S6.** Representative single cell-tracking video in ARK-1 cells treated with 100 µM H_2_O_2_.
